# Multimorbidity patterns and hospitalizations due to lower respiratory tract infections: a 15-year population-based cohort study

**DOI:** 10.1093/ageing/afag140

**Published:** 2026-05-19

**Authors:** Laura Franza, Susanna Gentili, Caterina Gregorio, Giorgi Beridze, Alessandra Marengoni, Stefania Maggi, Amaia Calderón-Larrañaga, Davide L Vetrano

**Affiliations:** PhD Program in Clinical and Experimental Medicine, Università degli studi di Modena and Reggio Emilia, Modena, Emilia-Romagna, Italy; Aging Research Center, Department of Neurobiology, Care Sciences and Society, Karolinska Institutet, Stockholm, Sweden; Aging Research Center, Department of Neurobiology, Care Sciences and Society, Karolinska Institutet, Stockholm, Sweden; Aging Research Center, Department of Neurobiology, Care Sciences and Society, Karolinska Institutet, Stockholm, Sweden; Aging Research Center, Department of Neurobiology, Care Sciences and Society, Karolinska Institutet, Stockholm, Sweden; Department of Clinical and Experimental Sciences, University of Brescia, Brescia, Italy; Neuroscience Institute, National Research Council, Padova, Lazio, Italy; Aging Research Center, Department of Neurobiology, Care Sciences and Society, Karolinska Institutet, Stockholm, Sweden; Stockholm Gerontology Research Center, Stockholm, Stockholm County, Sweden; Aging Research Center, Department of Neurobiology, Care Sciences and Society, Karolinska Institutet, Stockholm, Sweden; Stockholm Gerontology Research Center, Stockholm, Stockholm County, Sweden

**Keywords:** LRTIs, hospitalizations, multimorbidity, multimorbidity patterns, older people

## Abstract

**Background:**

Lower respiratory tract infections (LRTIs) are common among older adults and are associated with high healthcare utilization, particularly in those with multimorbidity (≥2 chronic conditions). We aimed to investigate the association between different multimorbidity patterns and LRTI-related hospitalizations.

**Materials and methods:**

We used data from the population-based Swedish National study on Aging and Care in Kungsholmen (SNAC-K). Latent class analysis was applied to participants with multimorbidity to identify multimorbidity patterns. A disease was considered to characterize a pattern if exclusivity was ≥25% or the observed/expected ratio was ≥2. Cox regressions were used to estimate hazard ratios (HRs) for the association between multimorbidity patterns and [1] LRTI-related hospitalizations and [2] all-cause 30-day readmission, compared with participants without multimorbidity.

**Results:**

Among the 3301 study participants, 2931 (87.5%) had multimorbidity, and over a median follow-up of 14 years, 412 (12.5%) were hospitalized with an LRTI and 152 (4.5%) experienced a 30-day readmission. Five multimorbidity patterns were identified. The *cardiometabolic* pattern showed the highest hazards for LRTI-related hospitalizations and 30-day readmissions, followed by the *psychiatric/respiratory* pattern. The *neuropsychiatric* pattern was associated with increased hazard of 30-day readmissions.

**Conclusions:**

Multimorbidity patterns are differentially associated with LRTI-related hospitalizations and 30-day readmissions, suggesting that the link between LRTIs and multimorbidity patterns is complex and reflects the specific burden of diverse multimorbidity patterns.

## Key points

- Lower respiratory tract infections (LRTIs) are common in the older population.
- People with multimorbidity are more severely affected by LRTIs, experiencing a higher risk of hospitalization and subsequent readmissions.
- Not all multimorbidity patterns are the same in increasing the patient’s risk of experiencing severe LRTIs.
- Identifying people at higher risk may allow us to enforce targeted preventive strategies, improving and personalizing patient care.

## Introduction

Lower respiratory tract infections (LRTIs), (e.g. pneumonia, bronchitis, bronchiolitis), can severely impact health, particularly in older adults [1]. These infections contribute to make these individuals frailer, and have complex clinical implications [2]. In older populations, LRTIs are among the leading causes of hospitalization and mortality, representing a major trigger for intense healthcare utilization [3–5]. LRTIs can partly be prevented through influenza and pneumococcal immunization in older adults. Momouris *et al.* reported that pneumococcal vaccination had a protective effect against LRTIs, particularly in those 50–84 years [6]; similar benefits have been observed for influenza vaccination [7]. The identification of older individuals at higher risk of LRTIs remains a priority to tailor preventive interventions [8].

In Sweden, the hospitalization rate for LRTIs is 23.0/1000 person-years, but for people over 80 years of age, this rate increases by 50% [9]. In Europe, it has been observed that hospitalization rates for LRTIs are lower overall (7.0/1000 person-years), but reach 30.0/1000 person-years in people 79 years of age and older [10].

People suffering from chronic conditions, particularly if older, are more likely to experience more severe LRTIs, thus needing to be hospitalized [11]. Research has focused on the association between respiratory comorbidity and LRTIs, but also conditions such as diabetes, chronic heart failure and cancer have been associated with higher rates of LRTIs and need for hospitalization [12].

People with multimorbidity, defined as the presence of at least two chronic conditions in the same individual [13, 14], are hospitalized up to six times more frequently due to an LRTI, which is also a major driver of in-hospital complications [12, 15, 16]. Older adults with multimorbidity face an increased risk of both LRTI-related hospitalization and readmission, compared to the general population [17, 18].

Multimorbidity affects up to 90% of individuals over 60 years old and is associated with complex pharmacological regimens [19], functional decline [20], reduced health-related quality of life [21] and depression [22]. Furthermore, multimorbidity increases the risk of hospitalization during acute illnesses such as LRTIs [22]. However, multimorbidity is not a single entity but rather occurs following patterns of chronic diseases, potentially influencing acute disease and functional outcomes [23–25]. However, the impact of specific multimorbidity patterns on LRTI-related hospitalizations and subsequent readmissions remains unclear.

This study aims to contribute filling this knowledge gap, evaluating how different multimorbidity patterns affect LRTI-related hospitalizations and 30-day all-cause readmissions in older adults.

## Materials and methods

### Study population

We performed a population-based cohort study, using data from the Swedish National study on Aging and Care in Kungsholmen (SNAC-K) [26]. SNAC-K is an ongoing prospective cohort study, and its population consists of individuals aged 60 or older, living at home or in institutions, on the island of Kungsholmen in central Stockholm (Sweden). The baseline data collection took place during 2001–4, for which the population was stratified based on age. Until age 78, participants are followed up every 6 years, and from age 78 onward, follow-up takes place every 3 years. At baseline, data from 3363 participants were collected, including sociodemographic characteristics, neuropsychological tests, physical examinations, medication use, disability and laboratory tests. Data from the Swedish National Patient Register (NPR) [27] was also included, providing information on hospitalizations and outpatient specialized care. Sixty-two SNAC-K participants withdrew their consent to link NPR data; thus, information was available for 3301 participants. Data from the Swedish Cause of Death Register [28] were also integrated. We analysed register data collected up to the 1^st^ of January 2017.

### Outcome definition

We evaluated two outcomes based on NPR data: LRTI-related hospitalizations and all-cause 30-day readmissions following an LRTI hospitalization episode. Time to LRTI-related hospitalization was defined as the time to the first event after the baseline SNAC-K interview date. Hospitalizations were defined as any admission involving at least one overnight stay. All-cause 30-day readmissions were defined as any hospitalization occurring within 30 days after discharge from the first hospitalization. We identified the causes of readmission, classifying them by International Classification of Diseases, 10th revision (ICD-10) code, as in Appendix S1. To identify LRTIs, we included all hospitalizations with a main diagnosis coded following International Classification of Diseases, 10th revision (ICD-10) [29] categories, among those listed in the supplementary materials (Appendix S2) [30].

### Chronic disease assessment

Chronic diseases, i.e. long-lasting conditions that cause lasting impairment or require sustained management, were identified through medical examinations conducted by SNAC-K physicians, participant and/or proxy self-report, instrumental and laboratory tests, use of medications and the NPR. Diagnoses were coded according to the ICD-10, and 918 chronic conditions were identified and classified into 60 chronic diseases [14] (Appendix S3).

### Covariates

Sociodemographic characteristics included age, sex, civil status and educational attainment. Age was analysed as a continuous variable, sex was classified as male/female and education as primary, secondary and university education or above. Civil status was categorized as partnered/married, single/divorced and widowed. Smoking habits were assessed and classified as past, current or never. Institutionalization, defined as living in a residential institution, was also evaluated as a binary categorical variable. Length of hospital stay (LoS) during the first hospitalization was evaluated as a continuous variable, measured in days, when analysing readmissions. Walking speed was evaluated as a dichotomic variable, using 0.8 m/s as a cutoff. Disability was evaluated through the presence of impairment in any activity of daily living (ADL). The score evaluates independence in bathing, dressing, toileting, transferring, continence, feeding and ranges from zero (no autonomy in any activity) to six (full autonomy in all activities) [31]. Any score under six was considered an indicator of impairment.

### Statistical analysis

Baseline characteristics were reported in terms of means and standard deviations for continuous variables; counts and percentages were used for categorical variables. Chi-square and *t*-test were used for testing differences in demographic and other characteristics based on being hospitalized or not for an LRTI.

To identify multimorbidity patterns, all participants with at least two chronic diseases were included in a latent class analysis (LCA). The optimal number of latent classes was determined through the Bayesian Information Criterion (BIC). Participants were assigned to the class for which they had the highest membership probability, allowing diseases to be shared across classes. A disease was considered to characterize a pattern if exclusivity was ≥25% or the observed/expected (O/E) ratio was ≥2, and patterns were labelled accordingly [32].

We performed a Cox regression to estimate hazard ratios (HRs) and 95% confidence intervals (CIs) for LRTI-related hospitalizations and all-cause 30-day readmissions, comparing individuals with different multimorbidity patterns to those without multimorbidity at baseline. The proportionality of the hazards was tested through Schoenfeld residuals test. The analysis was adjusted for age, sex, civil status, disability, walking speed and smoking. We stratified the analysis for age, sex, disability, walking speed and performed a sensitivity analysis on participants who were not institutionalized, as people who are institutionalized are likelier to have a higher morbidity burden, more severe cognitive impairment, to be more frail, which are all associated to more severe LRTIs and a higher likelihood to be hospitalized [33]. Follow-up time (in years) was calculated from study entry until the first LRTI hospitalization, 30-day readmission or until the end of the follow-up period (1 January 2017).

Kaplan–Meier survival curves were generated to estimate risks by multimorbidity pattern, and differences were tested using the log-rank test. Statistical significance was set at *P* < .05.

We created a heatmap to evaluate any association between seasonality and LRTI-associated hospitalizations and all-cause 30-day readmissions (Appendix S4).

To examine the cumulative risk of hospital readmission within 30, 60, 90 and 120 days following a first LRTI, we used Poisson regression models with a log link function. Adjusted predicted risks were estimated from the fitted models and plotted across follow-up time points (Appendix S5).

Statistical analyses were performed using R.4.4.1. The *survival* [34], *survminer* [35], *geepack* [36] and *poLCA* [37] packages were used to run the analyses.

## Results

Of the 3301 participants in the study, 64.9% were female and the average age was 74.8 (±11.2). A summary of participants’ sociodemographic characteristics, lifestyle habits and multimorbidity patterns can be found in Table 1.

**Table 1:** Baseline sample characteristics

Characteristic	No LRTI *n* (%) or mean ± SD	LRTI *n* (%) or mean ± SD	Overall *N* (%)or mean ± SD	*P*-value
Overall	2889 (87.5)	412 (12.5)	3301 (100)	
Age	74.2 ± 11.3	78.6 ± 9.5	74.8 ± 11.2	**<.001**
Sex (F)	1879 (65.5)	263 (60.6)	2142 (64.9)	**.028**
*Civil Status*				.3
Partnered/married	1324 (46.4)	175 (40.5)	1499 (45.4)	
Single/divorced	794 (27.8)	112 (25.9)	906 (27.4)	
Widowed	736 (25.8)	145 (33.6)	881 (26.6)	
*Education status*				**.015**
Primary education	476 (13.4)	103 (24.0)	579 (17.5)	
Secondary education	1413 (49.8)	215 (50.0)	1628 (49.3)	
University education	951 (33.5)	112 (26.0)	1063 (32.2)	
*Multimorbidity patterns*				**<.001**
No Multimorbidity	383 (13.4)	29 (6.7)	412 (12.5)	
Unspecific	1129 (39.2)	130 (30.0)	1259 (38.1)	
Sensory impairment/anaemia	480 (16.7)	87 (20.0)	567 (17.2)	
Psychiatric/respiratory	390 (13.6)	78 (18.0)	468 (14.2)	
Cardiometabolic	244 (8.5)	86 (19.8)	330 (10.0)	
Neuropsychiatric	241 (8.4)	24 (5.5)	265 (8.0)	
*Smoking status*				.6
Non-smoker	1335 (48.1)	187 (43.9)	1522 (46.1)	
Past smoker	1049 (37.8)	173 (40.6)	1222 (37.0)	
Current smoker	392 (14.1)	66 (15.5)	458 (13.9)	
Institutionalization	174 (6.1)	15 (3.5)	189 (5.7)	.10

Missingnacross variables: 15 smoking status, 15 social status, 15 education status. Abbreviations: F:female; SD: standard deviation.

Most participants had multimorbidity (2889, 87.5%). We identified five different multimorbidity patterns in this population: 1259 (38.1%) participants belonged to the *unspecific* pattern (i.e. no disease was overexpressed to characterized this pattern), 567 (17.2%) to the *sensory impairment/anaemia* pattern (the most prevalent diseases were Cataract lens disease Other eye disease), 468 (14.2%) to the *psychiatric/respiratory* pattern (the most prevalent diseases were Neurotic stress somatic disorder and Asthma), 330 (10.0%) to the *cardiometabolic* pattern (the most prevalent diseases were Bradycardias/conduction disease and Heart failure), and 265 (8.0%) to the *neuropsychiatric* pattern (the most prevalent diseases were Dementia and Other psychiatric behavioural disorders, i.e. organic psychiatric disorders and psychiatric disorders associated to substance use). Overall, the *psychiatric/respiratory* pattern was characterized by stress and mood disorders; besides the respiratory components, the *neuropsychiatric* pattern was characterized by behavioural disorders. A summary of diseases with O/E ≥2 and exclusivity ≥25% for each pattern is presented in Table 2.

**Table 2:** Diseases characterizing each multimorbidity pattern

Diseases per pattern	O/E	Exclusivity %	Prevalence %	Multimorbidity patterns (%)
NA				Unspecific pattern (38.0)
Cataract lens disease	3.80	75	24	Sensory impairment/anaemia pattern (17.2)
Other eye disease	3.50	69	20	
Glaucoma	3.45	68	22	
Blindness visual loss	2.61	51	13	
Anaemia	2.23	44	31	
Deafness hearing loss	2.07	41	27	
Neurotic stress somatic disorder	4.27	69	15	Psychiatric/respiratory pattern (14.1)
Asthma	3.53	57	24	
Depression mood disorder	3.33	54	35	
Dorsopathies	2.57	42	19	
Sleep disorder	2.47	40	6	
Osteoporosis	2.25	36	17	
COPD	2.23	36	13	
Oesophagus stomach duodenal disease	2.21	36	11	
Thyroid disease	2.03	33	24	
Bradycardias/conduction disease	6.27	71	13	Cardiometabolic pattern (9.9)
Heart failure	6.43	73	77	
Cardiac valve disease	5.11	58	14	
Other cardiovascular disease	4.84	55	19	
Atrial fibrillation	4.21	48	46	
Inflammatory arthropathies	2.83	32	13	
Ischemic heart disease	3.34	38	58	
Diabetes	2.69	30	27	
COPD	2.50	28	14	
Anaemia	2.25	25	31	
Cerebrovascular disease	2.22	25	20	
Dementia	8.09	75	88	Neuropsychiatric pattern (8.0)
Other psychiatric behavioural disorders	5.28	49	13	
Colitis-related disease	3.26	30	47	
Cerebrovascular disease	3.07	28	27	
Deafness hearing loss	2.81	26	37	
Blindness visual loss	2.79	26	14	

Exclusivity: number of patients with the disease in the pattern divided by the total number of participants with the disease; O/E (observed/expected) ratio: prevalence of a given disease within a pattern divided by its prevalence in the overall population.

**Figure 1:**
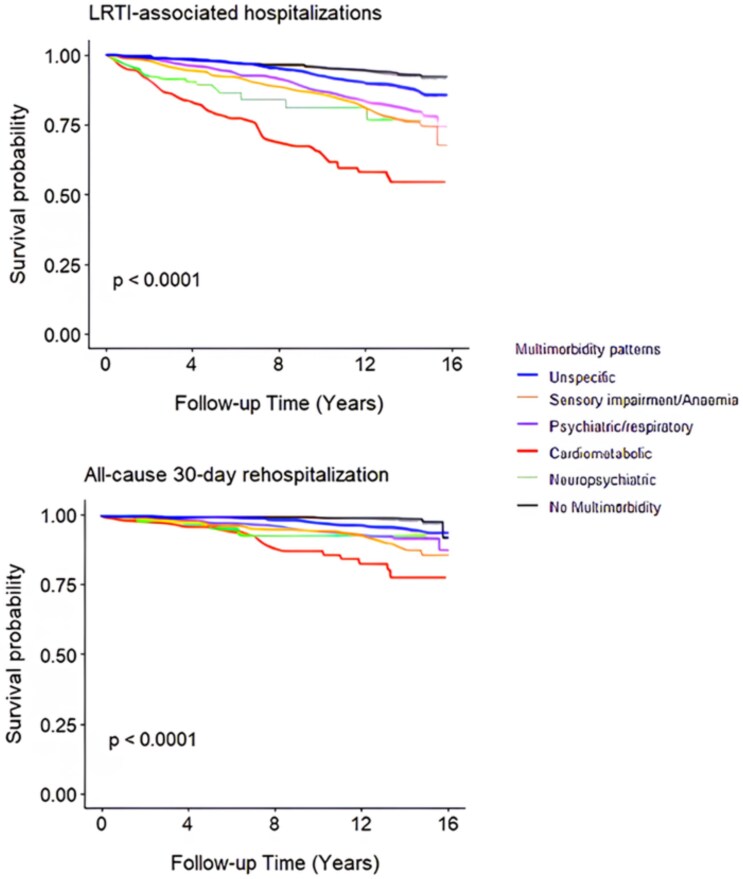
Survival curves for LRTI- associated hospitalizations and all-cause 30-day readmissions by multimorbidity pattern. Kaplan–Meier survival curves showing (top) LRTI-associated hospitalizations and (bottom) all-cause 30-day rehospitalization over 16 years of follow-up, stratified by multimorbidity patterns. Differences between groups were statistically significant (log-rank *P* < .0001).

Over a median follow-up of 14.0 years (inter-quartile range 1.6 years), 434 (13.0%) participants experienced at least one LRTI-related hospitalization and 152 (4.5%) had a 30-day readmission following discharge after an LRTI. 1617 (49.0%) participants died during the follow-up without experiencing an LRTI-related hospitalization. Figure 1 shows the survival curves for LRTI-related hospitalizations and 30-day re-admissions across multimorbidity patterns.

We evaluated if there was an association between seasonality and multimorbidity in LRTI-associated hospitalizations and all-cause 30-day readmissions, using a heatmap (Appendix S4): we observed some heterogeneity in both hospitalization and rehospitalization across multimorbidity patterns and calendar months, with evidence of seasonal fluctuations and consistently lower burden among individuals without multimorbidity.

Table 3 shows the incidence rates and HRs for the two outcomes of interest across different multimorbidity patterns, taking participants free from multimorbidity as the reference group. After adjusting by age, sex, education, civil status, disability, walking speed and smoking status, the *cardiometabolic* pattern, followed by the *psychiatric/respiratory* pattern, showed the highest hazards for both first hospitalization due to LRTI. The *cardiometabolic* and the *neuropsychiatric* patterns were associated with an increased hazard of 30-day readmission (Table 3).

**Table 3:** Cox regression for LRTI-associated-hospitalizations and 30-day readmissions (reference group: individuals without multimorbidity)

	LRTI-associated hospitalization			All-cause 30-day readmission		
	Hospitalized/total	Incidence rate	Cox model	Hospitalized/total	Incidence rate	Cox model
		Per 100 person-years	HR (95% CI)		Per 100 person-years	HR (95% CI)
Multimorbidity patterns						
No Multimorbidity	29/412	0.56	REF	6/412	0.11	REF
Unspecific	130/1259	0.90	1.09 (0.72; 1.64)	47/1259	0.32	1.33 (0.49; 3.58)
Sensory impairment/anaemia	87/567	1.84	1.26 (0.79; 2.00)	36/567	0.75	1.39 (0.48; 4.02)
Psychiatric/respiratory	78/468	1.55	**1.85 (1.19; 2.88)**	30/468	0.58	1.89 (0.68; 5.22)
Cardiometabolic	86/330	4.56	**2.87 (1.80; 4.59)**	27/330	1.36	**3.08 (1.06; 8.92)**
Neuropsychiatric	24/265	2.79	1.68 (0.87; 3.21)	6/265	0.68	**4.29 (1.06; 17.44)**

Abbreviations: CI: confidence interval; HR: hazard ratio.

Models were adjusted for sex, age, education status, civil status, smoking status, walking speed and disability and LoS, for all-cause 30-day readmissions. HRs in bold indicate statistical significance.

Among those readmitted, about 50% re-entered because of a respiratory infection (Appendix S1): while it is possible that people who were readmitted developed new infections between hospitalizations, it is also possible that patients were readmitted because the first infection had not fully resolved in the first place [38].

We performed a stratified analysis by age—using 78 years as the cut-off [39]-, by sex, by walking speed, by disability and by institutionalization. The *cardiometabolic* and the *psychiatric/respiratory* patterns were significant in all groups, except for older participants, those who had a worse walking speed and those who were institutionalized. In general, in these subgroups, no multimorbidity pattern was associated with an increased hazard of LRTI-associated hospitalizations. Younger, male and impaired participants showed an association between the *neuropsychiatric* pattern and LRTI-associated hospitalizations (Appendix S6). A similar trend was observed in the hazard of all-cause 30-day readmissions: participants who were older and slower did not show any association with multimorbidity patterns; also, women did not present any association with multimorbidity. Younger participants belonging to the *psychiatric/respiratory* and the *sensory impairment/anaemia* patterns showed an increased hazard of readmission (Table 3).

We evaluated whether LoS is associated to an increased hazard of rehospitalization for a respiratory infection, but the sensitivity analysis we performed did not confirm our hypothesis; no pattern was associated to an increased hazard of 30-day readmission in this subgroup (Appendix S7).

In a Poisson regression model (Appendix S5), using generalized estimating equations (GEE), the cumulative risk of hospital readmission following a first LRTI decreased progressively across follow-up time points (30, 60, 90 and 120 days). The *psychiatric/respiratory* pattern showed the highest risk of readmission at all times, yet, over time, trajectories appeared parallel across groups, suggesting that absolute risk differs by multimorbidity pattern, but temporal decline in risk is similar across patterns.

## Discussion

In our study, older adults with specific multimorbidity patterns (i.e. *cardiometabolic, psychiatric/respiratory* and *neuropsychiatric*) were at increased risk of LRTI-related hospitalizations and all-cause 30-day readmissions.

The role of multimorbidity in influencing LRTI-related hospitalizations and 30-day readmissions has been previously investigated. In a study by Anza-Ramirez et al., multimorbidity increased the risk of developing acute infectious conditions, particularly LRTIs [40, 41], and was associated with an increased need for hospitalization [42]. Likewise, in a study by Nguyen et al. [18], the presence of a higher comorbidity burden, assessed through the Charlson index, was associated with a higher risk of readmission within 30 days following a LRTI. People with multimorbidity are also more likely to be hospitalized, be admitted to the ER, or decease during the 90 days following a LRTI [17]. Yet, previous studies have not examined the role of different multimorbidity patterns, despite evidence from many studies indicating that such patterns are informative with respect to one’s prognosis in diverse contexts [43]. In our study, multimorbidity was confirmed to increase the risk of both hospitalization due to an LRTI and all-cause 30-day readmissions, but not all multimorbidity patterns conferred the same level of risk.

In our study, belonging to the *cardiometabolic* pattern was significantly associated with LRTI-related hospitalizations. The most prevalent conditions were bradycardia and heart failure, the latter of which has previously been linked to poorer outcomes in patients with LRTIs [44, 45]. Diabetes was also highly prevalent, and its association with more severe LRTIs is well established, likely due to immune dysfunction [46]. The *cardiometabolic* pattern was also significantly associated with all-cause 30-day readmissions: other studies showed that people belonging to the *cardiometabolic* pattern are at higher risk of recurrent hospitalizations following acute events [15, 47]. While evidence remains inconclusive, some authors have suggested that the *cardiometabolic* pattern may impair immune responses, increasing susceptibility to infections such as LRTIs [48].

In the *psychiatric/respiratory* pattern, the two most prevalent conditions were asthma and neurotic stress and somatic disorders (i.e. mostly anxiety), which have been identified as risk factors for more severe LRTIs, particularly in older people [49, 50]. Psychiatric disorders, especially neurotic stress and somatic disorders, have been associated with physiological dysfunctions [51], including immune system impairment, which may increase vulnerability to infections [52]. Mental illness has also been shown to substantially increase healthcare utilization associated with an LRTI [53]. COPD was another common condition in this pattern and is likely to contribute to the association with LRTI-related hospitalizations [54]. It is worth noting that poor adherence to COPD medication, which is common in this population, has been associated with an increased risk of hospitalizations [55]. People belonging to this pattern were not more likely to be readmitted during the following 30 days after the first admission, compared to people belonging to other multimorbidity patterns. While it has been reported that people suffering from chronic respiratory disorders are more likely to be hospitalized again during the 30-days following an LRTI [56, 57], it has also been observed that specific strategies can be employed to reduce their readmission risk and in Sweden, specialized COPD clinics have proven effective in reducing exacerbations, which could explain our findings [58].

The *neuropsychiatric* pattern was significantly associated with all-cause 30-day readmissions. Dementia was the most prevalent condition in this pattern and is a well-known risk factor for aspiration pneumonia. Moreover, dementia can impair the ability to recognize or report symptoms, leading to delayed care. People with dementia also present with poorer oral hygiene, which is in itself a risk factor for pneumonia [59, 60].

In our stratified analysis, we observed that participants who were older and in poorer physical health did not present an increased hazard of hospitalization or readmissions associated to any multimorbidity pattern, which is consistent with the observation that, with age and declining physical health, multimorbidity becomes less accurate in predicting health trajectories [61]. On the other hand, in younger participants, other patterns were significantly associated to an increased hazard of 30-day readmission: the *sensory impairment/anaemia* pattern, for instance, emerged as significantly associated with all-cause 30-day readmissions. The most prevalent condition in this pattern was visual impairment, which has been linked with an increased risk of 30-day readmissions following an acute event [62], although the underlying mechanisms remain unclear. Also, anaemia has been associated with increased frailty, which in turn could increase hospital readmission rates [63, 64]. Younger participants and those who had a higher walking speed belonging to the *psychiatric/respiratory* pattern had a higher hazard of all-cause 30-day readmission. One explanation is that younger participants belonging to this pattern may present with more severe forms of respiratory diseases, associated to an increased risk of readmission following an LRTI [12]. It is also possible that this group of patients has a more intense social life, increasing the possibility of being exposed to infections [65].

The *psychiatric/respiratory* pattern is also the most strongly associated to a cumulative admission risk during the 120 days following the first LRTI-hospitalization (Appendix S5), consistent with literature [66, 67]: people with underlying respiratory conditions and hospitalized due to an LRTI are particularly vulnerable post-discharge and have a high risk of developing conditions requiring a new hospitalization, worsening their overall health and burdening health systems [68].

We also decided to evaluate whether the association between multimorbidity patterns, hospitalizations and readmissions could be influenced by seasonality (Appendix S4): we observed seasonal variation, with higher rates of hospitalizations and readmissions, particularly in the winter period, which has been described in literature [69, 70].

Overall, being able to identify multimorbidity patterns associated with a higher risk of hospitalization and readmission due to an LRTI, could have some practical implications: identifying those who are at a higher risk of hospitalization or readmission could allow us to better tailor interventions during their hospital stays; also, this population could be the object of a precise prevention strategy, perhaps allowing to reduce hospitalizations in the first place.

## Strengths and limitations

The SNAC-K cohort offers long-term follow-up, with participants observed for up to 15 years, which enabled detecting both LRTI-related hospitalizations and 30-day readmissions. SNAC-K includes extensive in-person evaluations of chronic conditions. Another strength of the study is the link to national registers, which ensured accurate identification of outcomes. However, the study population is relatively homogenous and generally healthier and wealthier than national averages. Also, we were unable to capture LRTIs not requiring hospitalization, preventing the assessment of the impact of multimorbidity on milder infections. Finally, information on the use of preventive measures such as vaccinations was not available and could therefore not be considered, yet the Swedish population, particularly older persons, have high vaccination rates, which can promote herd immunity [71].

## Conclusions

LRTIs are a common cause of hospitalization and readmission in the older population. Although multimorbidity is known to increase the risk of hospital care due to acute events, the role of specific multimorbidity patterns in increasing the risk of LRTIs has not been previously examined. The *cardiometabolic* and *psychiatric/respiratory* patterns were associated with an increased risk of hospitalization; the *cardiometabolic* pattern maintained the association with all-cause 30-day readmissions, which it shared with the *neuropsychiatric* pattern. These findings highlight the importance of considering multimorbidity patterns when evaluating health outcomes, even after accounting for sociodemographic and other clinical factors, particularly in younger and healthier people. Future research should clarify the mechanisms underlying these associations, determine whether other acute conditions show distinct relationships with multimorbidity patterns and explore how these patterns can be used to guide the personalization of LRTI prevention strategies.

## Supplementary Material

Supplementary_materials_afag140
