# Supplementary material for: Multimorbidity patterns and hospitalizations due to lower respiratory tract infections: a 15-year population-based cohort study
Source: Age Ageing. 2026 May 19;55(5):afag140. doi: 10.1093/ageing/afag140 (PMC13184961; doi:10.1093/ageing/afag140)
Supplement: Supplementary_materials_afag140 [file supplementary_materials_afag140.docx]

**Multimorbidity patterns and hospitalizations due to lower respiratory tract infections: a 15-year population-based cohort study**

Table of contents

[Appedix 1. All-cause 30-day rehospitalizations per multimorbidity pattern 2](#__RefHeading___Toc12035_2766583487)

[Appendix 2. LRTI identification 4](#__RefHeading___Toc12037_2766583487)

[Appendix 3. ICD-10 codes, clinical and drug-related* parame^t^ers used in SNAC-K for specific chronic conditions. 5](#__RefHeading___Toc12039_2766583487)

[Appendix 4. Association between seasonality and multimorbidity patterns in LRTI-associated hospitalizations and all-cause 30-day readmissions 37](#__RefHeading___Toc12041_2766583487)

[Appendix 5. Hazard of hospital readmission within 120 days following first LRTI across multimorbidity patterns 39](#__RefHeading___Toc12043_2766583487)

[Appendix 6. Sensitivity analysis per age, sex, walking speed, disability, institutionalization and LRTI-associated 30-day rehospitalization 40](#__RefHeading___Toc12045_2766583487)

[Appendix 7. Analysis on LRTI-associated 30-day readmissions 41](#__RefHeading___Toc12047_2766583487)

[43](#__RefHeading___Toc12049_2766583487)

[43](#__RefHeading___Toc12051_2766583487)

[43](#__RefHeading___Toc12053_2766583487)

[43](#__RefHeading___Toc12055_2766583487)

[45](#__RefHeading___Toc12057_2766583487)

[45](#__RefHeading___Toc12059_2766583487)

[45](#__RefHeading___Toc12061_2766583487)

# **Appedix 1****. All-cause 30-day rehospitalizations per multimorbidity pattern**

| Causes of rehospitalization (ICD-10 codes) | Multimorbidity patterns | | | | | | Overall  N (%) |
| --- | --- | --- | --- | --- | --- | --- | --- |
|  | No Multimorbidity  n (%) | Unspecific  n (%) | Sensory impairment/  Anaemia  n (%) | Psychiatric/  Respiratory  n (%) | Cardiometabolic  n (%) | Neuropsychiatric  n (%) |  |
| Overall | 6 (3.95) | 47 (30.92) | 36 (23.68) | 30 (19.74) | 27 (17.76) | 6 (3.95) | 152 (100) |
| Influenza and pneumonia (J13.9, J14.9, J15.9, J18.0, J18.9) | 5 (83.3) | 24 (51.06) | 21 (58.33) | 10 (33.33) | 12 (44.44) | 3 (50) | 75 (49.34) |
| Heart disease (I20.9, I21.4, I21.4X, I48.9, I50.9) |  | 3 (6.38) | 3 (8.33) | 1 (3.33) | 6 (22.22) |  | 13 (8.55) |
| Other diseases of the respiratory system (J06.9, J44.0, J44.1, J44.9, J86.9, J90.9, J96.0, S22.3) | 1 (16.67) | 2 (4.26) | 1 (2.78) | 6 (20.00) | 2 (7.40) |  | 12 (7.89) |
| Neoplasms (C20.9, C34.9, D37.7, D38.1, D46.2, D33.7) |  | 2 (4.26) | 1 (2.78) | 2 (6.67) | 2 (7.71) |  | 9 (5.92) |
| Muscular-skeletal disorders (I10.9, M43.6, M48.0, M48.0, M48.5, S72.0) |  | 2 (4.26) | 2 (5.56) | 3 (10.00) | 1 (3.70) |  | 8 (5.26) |
| Nervous system disorders (A80.1, F03.9, F05.0, G30.1, G30.8, G31.8, I61.6, I63.9) |  | 4 (8.52) | 1 (2.78) | 1 (3.33) |  | 2 (33.3) | 8 (5.26) |
| Gastrointestinal disorders (A04.7, K08.8, K22.0, K56.2, R11.9, R17.9) |  | 2 (4.26) | 1 (2.78) | 1 (3.33) | 2 (5.40) | 1 (16.67) | 7 (4.61) |
| Other disorders (R50.9, R70.0, R91.9, T46.0, Z47.9, Z51.1, Z51.4) |  | 2 (4.26) | 3 (8.34) | 1 (3.33) | 1 (3.70) |  | 7 (4.61) |
| Vascular-metabolic disorders (E83.5, E86.9, E87.8, I70.2, I71.4, I74.8) |  | 3 (6.38) |  | 3 (10.00) | 1 (3.70) |  | 7 (4.61) |
| Genito-urinary system disorders (N17.9, N39.0, N40.9, N81.5) |  | 3 (6.39) |  | 1 (3.33) |  |  | 4 (2.63) |
| Other bacterial diseases (A31.0, A46.9, B59.9) |  |  | 2 (5.56) | 1 (3.33) |  |  | 3 (1.97) |

A04.7, Enterocolitis due to Clostridium difficile; A31.0, Pulmonary mycobacterial infection; A46.9, Erysipelas; A81.0, Creutzfeldt-Jakob disease, unspecified; B59.9, Pneumocystosis; C20.9, Malignant neoplasm of rectum; C34.9, Malignant neoplasm of bronchus or lung, unspecified; D33.7, Benign neoplasm of other specified parts of central nervous system; D37.7, Neoplasm of uncertain behavior of other digestive organs; D38.1, Neoplasm of uncertain behavior of trachea, bronchus and lung; D46.2, Refractory anemia with excess of blasts; E83.5, Unspecified disorder of calcium metabolism; E869, Volume depletion, unspecified; E87.8, Other disorders of electrolyte and fluid balance, not elsewhere classified; F03.9, Unspecified dementia without behavioral disturbance; F05.0, Delirium not superimposed on dementia, so described; G30.1, Alzheimer's disease with late onset; G30.8, Other Alzheimer's disease; G31.8, Other specified degenerative diseases of nervous system; I10.9, Essential (primary) hypertension; I20.9, Angina pectoris, unspecified; I21.4, Non-ST elevation (NSTEMI) myocardial infarction; I21.4X, Non-ST elevation (NSTEMI) myocardial infarction; I48.9, Atrial fibrillation and atrial flutter, unspecified; I50.9, Heart failure, unspecified; I61.6, Nontraumatic intracerebral hemorrhage, multiple localized; I63.9, Cerebral infarction, unspecified; I70.2, Atherosclerosis of arteries of extremities; I71.4, Abdominal aortic aneurysm, without rupture; I74.8, Embolism and thrombosis of other arteries; J06.9, Acute upper respiratory infection, unspecified; J13.9, Pneumonia due to Streptococcus pneumoniae; J14.9, Pneumonia due to Hemophilus influenzae; J15.9, Bacterial pneumonia, unspecified; J18.0, Bronchopneumonia, unspecified organism; J18.9, Pneumonia, unspecified; J20.9, Acute bronchitis, unspecified; J44.0, Chronic obstructive pulmonary disease with acute lower respiratory infection; J44.1, Chronic obstructive pulmonary disease with acute exacerbation; J44.9, Chronic obstructive pulmonary disease, unspecified; J86.9, Pyothorax without fistula; J90.9, Pleural effusion, not elsewhere classified; J96.0, Acute respiratory failure; K08.8, Other specified disorders of teeth and supporting structures; K22.0, Achalasia of cardia; K56.2, Volvulus; M19.0, Primary arthrosis of other joints; M43.6, Torticollis; M48.0, Spinal stenosis; M48.5, Collapsed vertebra, not elsewhere classified; N17.9, Acute renal failure, unspecified; N39.0, Urinary tract infection, site not specified; N40.9, Hyperplasia of prostate; N81.5, Vaginal enterocele; R11.9, Nausea and vomiting; R17.9, Hyperbilirubinaemia without mention of jaundice, not elsewhere classified; R50.9, Fever, unspecified; R70.0, Elevated erythrocyte sedimentation rate; R91.9, Abnormal findings on diagnostic imaging of lung; S22.3, Fracture of rib; S72.0, Fracture of neck of femur; T46.0, Poisoning by cardiac-stimulant glycosides and drugs of similar action; Z47.9, Orthopaedic follow-up care, unspecified; Z51.1, Encounter for antineoplastic chemotherapy; Z51.4, Preparatory care for subsequent treatment, not elsewhere classified.

# **Appendix 2****. LRTI identification**

| ICD-10 Code | Description |
| --- | --- |
| J09 | Influenza due to certain identified influenza viruses |
| J10 | Influenza due to other identified influenza virus |
| J11 | Influenza due to unidentified influenza virus |
| J12 | Viral pneumonia, not elsewhere classified |
| J13 | Pneumonia due to Streptococcus pneumoniae |
| J14 | Pneumonia due to Hemophilus influenzae |
| J15 | Bacterial pneumonia, not elsewhere classified |
| J16 | Pneumonia due to other infectious organisms, not elsewhere classified |
| J17 | Pneumonia in diseases classified elsewhere |
| J18 | Pneumonia, unspecified organism |
| J20 | Acute bronchitis |
| J21 | Acute bronchiolitis |
| J22 | Unspecified acute lower respiratory infection |

# **Appendix 3****. ICD-10 codes, clinical and drug-related^*^ parameters used in SNAC-K for specific chronic conditions.**

| **Condition** | **Included ICD-10 codes** | **Clinical and drug-related parameters** |
| --- | --- | --- |
| **Allergy** | J301 Allergic rhinitis due to pollen  J302 Other seasonal allergic rhinitis  J303 Other allergic rhinitis  J304 Allergic rhinitis, unspecified  J450 Predominantly allergic asthma  K522 Allergic and dietetic gastroenteritis and colitis  L20 Atopic dermatitis  L23 Allergic contact dermatitis  L500 Allergic urticaria  Z516 Desensitization to allergens |  |
| **Anemia** | D50 Iron deficiency anaemia  D51 Vitamin B12 deficiency anaemia  D52 Folate deficiency anaemia  D53 Other nutritional anaemias  D55 Anaemia due to enzyme disorders  D56 Thalassaemia  D57 Sickle-cell disorders  D58 Other hereditary haemolytic anaemias  D59 Acquired haemolytic anaemia  D60 Acquired pure red cell aplasia [erythroblastopenia]  D61 Other aplastic anaemias  D63 Anaemia in chronic diseases classified elsewhere  D64 Other anaemias | Hemoglobin <13 g/dl in men and <12 g/dl in women^1^  Use of iron preparations (B03A) or other antianemic preparations (B03XA) |
| **Asthma** | J45 Asthma | Use of leukotriene receptor antagonists (R03DC) or antiallergic agents, excl. corticosteroids (R03BC) |
| **Atrial fibrillation** | I48 Atrial fibrillation and flutter | Discrete P wave undetectable and irregular ventricular rate (12-lead electrocardiogram) |
| **Autoimmune diseases** | I731 Thromboangiitis obliterans [Buerger]  L10 Pemphigus  L12 Pemphigoid  L40 Psoriasis  L41 Parapsoriasis  L93 Lupus erythematosus  L94 Other localized connective tissue disorders  L95 Vasculitis limited to skin, not elsewhere classified  M30 Polyarteritis nodosa and related conditions  M31 Other necrotizing vasculopathies  M32 Systemic lupus erythematosus  M33 Dermatopolymyositis  M34 Systemic sclerosis  M35 Other systemic involvement of connective tissue  M36 Systemic disorders of connective tissue in diseases classified elsewhere | Use of antipsoriatics (D05) |
| **Blindness, visual impairment** | H54 Visual impairment including blindness (binocular or monocular)  Z442 Fitting and adjustment of artificial eye  Z970 Presence of artificial eye | Unable to see the physician at a close distance with or without aid (assessed by a nurse) |
| **Blood and blood organ forming diseases** | D66 Hereditary factor VIII deficiency  D67 Hereditary factor IX deficiency  D68 Other coagulation defects  D69 Purpura and other haemorrhagic conditions  D71 Functional disorders of polymorphonuclear neutrophils  D720 Genetic anomalies of leukocytes  D730 Hyposplenism  D731 Hypersplenism  D732 Chronic congestive splenomegaly  D74 Methaemoglobinaemia  D750 Familial erythrocytosis  D761 Haemophagocytic lymphohistiocytosis  D763 Other histiocytosis syndromes  D77 Other disorders of blood and blood-forming organs in diseases classified elsewhere  D80 Immunodeficiency with predominantly antibody defects  D81 Combined immunodeficiencies  D82 Immunodeficiency associated with other major defects  D83 Common variable immunodeficiency  D84 Other immunodeficiencies  D86 Sarcoidosis  D89 Other disorders involving the immune mechanism, not elsewhere classified |  |
| **Bradycardias and conduction diseases** | I441 Atrioventricular block, second degree  I442 Atrioventricular block, complete  I443 Other and unspecified atrioventricular block  I453 Trifascicular block  I455 Other specified heart block  Z950 Presence of cardiac pacemaker | Presence of a cardiac pacemaker (12-lead electrocardiogram) |
| **Cardiac valve diseases** | I05 Rheumatic mitral valve diseases  I06 Rheumatic aortic valve diseases  I07 Rheumatic tricuspid valve diseases  I08 Multiple valve diseases  I091 Rheumatic diseases of endocardium, valve unspecified  I098 Other specified rheumatic heart diseases  I34 Nonrheumatic mitral valve disorders  I35 Nonrheumatic aortic valve disorders  I36 Nonrheumatic tricuspid valve disorders  I37 Pulmonary valve disorders  I38 Endocarditis, valve unspecified  I390 Mitral valve disorders in diseases classified elsewhere  I391 Aortic valve disorders in diseases classified elsewhere  I392 Tricuspid valve disorders in diseases classified elsewhere  I393 Pulmonary valve disorders in diseases classified elsewhere  I394 Multiple valve disorders in diseases classified elsewhere  Q22 Congenital malformations of pulmonary and tricuspid valves  Q23 Congenital malformations of aortic and mitral valves  Z952 Presence of prosthetic heart valve  Z953 Presence of xenogenic heart valve  Z954 Presence of other heart-valve replacement |  |
| **Cataract and other lens diseases** | H25 Senile cataract  H26 Other cataract  H27 Other disorders of lens  H28 Cataract and other disorders of lens in diseases classified elsewhere  Q12 Congenital lens malformations  Z961 Presence of intraocular lens |  |
| **Cerebrovascular disease** | G45 Transient cerebral ischaemic attacks and related syndromes  G46 Vascular syndromes of brain in cerebrovascular diseases  I60 Subarachnoid haemorrhage  I61 Intracerebral haemorrhage  I62 Other nontraumatic intracranial haemorrhage  I63 Cerebral infarction  I64 Stroke, not specified as haemorrhage or infarction  I67 Other cerebrovascular diseases  I69 Sequelae of cerebrovascular disease |  |
| **Chromosomal abnormalities** | Q90 Down syndrome  Q91 Edwards syndrome and Patau syndrome  Q92 Other trisomies and partial trisomies of the autosomes, not elsewhere classified  Q93 Monosomies and deletions from the autosomes, not elsewhere classified  Q95 Balanced rearrangements and structural markers, not elsewhere classified  Q96 Turner syndrome  Q97 Other sex chromosome abnormalities, female phenotype, not elsewhere classified  Q98 Other sex chromosome abnormalities, male phenotype, not elsewhere classified  Q99 Other chromosome abnormalities, not elsewhere classified |  |
| **Chronic infectious diseases** | A15 Respiratory tuberculosis, bacteriologically and histologically confirmed  A16 Respiratory tuberculosis, not confirmed bacteriologically or histologically  A17 Tuberculosis of nervous system  A18 Tuberculosis of other organs  A19 Miliary tuberculosis  A30 Leprosy [Hansen disease]  A31 Infection due to other mycobacteria  A50 Congenital syphilis  A52 Late syphilis  A53 Other and unspecified syphilis  A65 Nonvenereal syphilis  A66 Yaws  A67 Pinta [carate]  A692 Lyme disease  A81 Atypical virus infections of central nervous system  B20 Human immunodeficiency virus [HIV] disease resulting in infectious and parasitic diseases  B21 Human immunodeficiency virus [HIV] disease resulting in malignant neoplasms  B22 Human immunodeficiency virus [HIV] disease resulting in other specified diseases  B23 Human immunodeficiency virus [HIV] disease resulting in other conditions  B24 Unspecified human immunodeficiency virus [HIV] disease  B381 Chronic pulmonary coccidioidomycosis  B391 Chronic pulmonary histoplasmosis capsulati  B401 Chronic pulmonary blastomycosis  B572 Chagas disease (chronic) with heart involvement  B573 Chagas disease (chronic) with digestive system involvement  B574 Chagas disease (chronic) with nervous system involvement  B575 Chagas disease (chronic) with other organ involvement  B65 Schistosomiasis [bilharziasis]  B92 Sequelae of leprosy  B94 Sequelae of other and unspecified infectious and parasitic diseases  J65 Pneumoconiosis associated with tuberculosis  M863 Chronic multifocal osteomyelitis  M864 Chronic osteomyelitis with draining sinus  M865 Other chronic haematogenous osteomyelitis  M866 Other chronic osteomyelitis | Use of drugs for treatment of tuberculosis excluding cycloserine, rifampicine, rifamicyne and hydrazides (J04A excl. J04AB01, J04AB02, J04AB03 and J04AC) |
| **Chronic kidney diseases** | I120 Hypertensive renal disease with renal failure  I130 Hypertensive heart and renal disease with (congestive) heart failure  I131 Hypertensive heart and renal disease with renal failure  I132 Hypertensive heart and renal disease with both (congestive) heart failure and renal failure  I139 Hypertensive heart and renal disease, unspecified  N01 Rapidly progressive nephritic syndrome  N03 Chronic nephritic syndrome  N04 Nephrotic syndrome  N05 Unspecified nephritic syndrome  N07 Hereditary nephropathy, not elsewhere classified  N08 Glomerular disorders in diseases classified elsewhere  N11 Chronic tubulo-interstitial nephritis  N183 Chronic kidney disease, stage 3  N184 Chronic kidney disease, stage 4  N185 Chronic kidney disease, stage 5  N189 Chronic kidney disease, unspecified  Q60 Renal agenesis and other reduction defects of kidney  Q611 Polycystic kidney, autosomal recessive  Q612 Polycystic kidney, autosomal dominant  Q613 Polycystic kidney, unspecified  Q614 Renal dysplasia  Q615 Medullary cystic kidney  Q618 Other cystic kidney diseases  Q619 Cystic kidney disease, unspecified  Z905 Acquired absence of kidney  Z940 Kidney transplant status | Glomerular filtration rate <60 ml/min/1.73m2 (assessed using the CKD-EPI equation)^2^ |
| **Chronic liver diseases** | B18 Chronic viral hepatitis  K70 Alcoholic liver disease  K713 Toxic liver disease with chronic persistent hepatitis  K714 Toxic liver disease with chronic lobular hepatitis  K715 Toxic liver disease with chronic active hepatitis  K717 Toxic liver disease with fibrosis and cirrhosis of liver  K721 Chronic hepatic failure  K73 Chronic hepatitis, not elsewhere classified  K74 Fibrosis and cirrhosis of liver  K753 Granulomatous hepatitis, not elsewhere classified  K754 Autoimmune hepatitis  K758 Other specified inflammatory liver diseases  K761 Chronic passive congestion of liver  K766 Portal hypertension  K767 Hepatorenal syndrome  K778 Liver disorders in other diseases classified elsewhere  Q446 Cystic disease of liver  Z944 Liver transplant status |  |
| **Chronic pancreas, biliary tract and gallbladder diseases** | K800 Calculus of gallbladder with acute cholecystitis  K801 Calculus of gallbladder with other cholecystitis  K802 Calculus of gallbladder without cholecystitis  K808 Other cholelithiasis  K811 Chronic cholecystitis  K86 Other diseases of pancreas  Q440 Agenesis, aplasia and hypoplasia of gallbladder  Q441 Other congenital malformations of gallbladder  Q442 Atresia of bile ducts  Q443 Congenital stenosis and stricture of bile ducts  Q444 Choledochal cyst  Q445 Other congenital malformations of bile ducts  Q450 Agenesis, aplasia and hypoplasia of pancreas | Use of multienzymes (lipase, protease etc.) (A09AA02) |
| **Chronic ulcer of the skin** | I830 Varicose veins of lower extremities with ulcer  I832 Varicose veins of lower extremities with both ulcer and inflammation  L89 Decubitus ulcer and pressure area  L97 Ulcer of lower limb, not elsewhere classified  L984 Chronic ulcer of skin, not elsewhere classified |  |
| **Colitis and related diseases** | K520 Gastroenteritis and colitis due to radiation  K528 Other specified noninfective gastroenteritis and colitis  K551 Chronic vascular disorders of intestine  K552 Angiodysplasia of colon  K572 Diverticular disease of large intestine with perforation and abscess  K573 Diverticular disease of large intestine without perforation or abscess  K574 Diverticular disease of both small and large intestine with perforation and abscess  K575 Diverticular disease of both small and large intestine without perforation or abscess  K578 Diverticular disease of intestine, part unspecified, with perforation and abscess  K579 Diverticular disease of intestine, part unspecified, without perforation or abscess  K58 Irritable bowel syndrome  K590 Constipation  K592 Neurogenic bowel, not elsewhere classified  K62 Other diseases of anus and rectum  K634 Enteroptosis  K64 Haemorrhoids and perianal venous thrombosis | Use of drugs for constipation (A06A) |
| **COPD, Emphysema, Chronic Bronchitis** | J41 Simple and mucopurulent chronic bronchitis  J42 Unspecified chronic bronchitis  J43 Emphysema  J44 Other chronic obstructive pulmonary disease  J47 Bronchiectasis | Use of anticholinergics (R03BB) |
| **Deafness, hearing impairment** | H80 Otosclerosis  H90 Conductive and sensorineural hearing loss  H911 Presbycusis  H913 Deaf mutism, not elsewhere classified  H919 Hearing loss, unspecified  Q16 Congenital malformations of ear causing impairment of hearing  Z453 Adjustment and management of implanted hearing device  Z461 Fitting and adjustment of hearing aid  Z962 Presence of otological and audiological implants  Z974 Presence of external hearing-aid | Unable to hear the interviewer’s voice at a normal volume (assessed by a nurse) |
| **Dementia** | F00 Dementia in Alzheimer disease  F01 Vascular dementia  F02 Dementia in other diseases classified elsewhere  F03 Unspecified dementia  F051 Delirium superimposed on dementia  G30 Alzheimer disease  G31 Other degenerative diseases of nervous system, not elsewhere classified | Diagnostic and Statistical Manual of Mental Disorders, Third Edition, Revised^3^ (assessed by two different physicians, and a third one in case of disagreement)  Use of anticholinesterases (N06DA) or memantine (N06DX01) |
| **Depression and mood diseases** | F30 Manic episode  F31 Bipolar affective disorder  F32 Depressive episode  F33 Recurrent depressive disorder  F34 Persistent mood [affective] disorders  F38 Other mood [affective] disorders  F39 Unspecified mood [affective] disorder  F412 Mixed anxiety and depressive disorder |  |
| **Diabetes** | E10 Insulin-dependent diabetes mellitus  E11 Non-insulin-dependent diabetes mellitus  E13 Other specified diabetes mellitus  E14 Unspecified diabetes mellitus  E891 Postprocedural hypoinsulinaemia | Glycated hemoglobin (A1C) ≥6.5%^4^  Use of antidiabetics (A10) |
| **Dorsopathies** | M40 Kyphosis and lordosis  M41 Scoliosis  M42 Spinal osteochondrosis  M43 Other deforming dorsopathies  M47 Spondylosis  M48 Other spondylopathies  M49 Spondylopathies in diseases classified elsewhere  M50 Cervical disc disorders  M51 Other intervertebral disc disorders  M53 Other dorsopathies, not elsewhere classified  Q675 Congenital deformity of spine  Q761 Klippel-Feil syndrome  Q764 Other congenital malformations of spine, not associated with scoliosis |  |
| **Dyslipidemia** | E78 Disorders of lipoprotein metabolism and other lipidaemias | Serum total cholesterol ≥6.22 mmol/L^5^ |
| **Ear, nose, throat diseases** | H604 Cholesteatoma of external ear  H661 Chronic tubotympanic suppurative otitis media  H662 Chronic atticoantral suppurative otitis media  H663 Other chronic suppurative otitis media  H701 Chronic mastoiditis  H71 Cholesteatoma of middle ear  H731 Chronic myringitis  H741 Adhesive middle ear disease  H810 MÚniÞre disease  H831 Labyrinthine fistula  H832 Labyrinthine dysfunction  H95 Postprocedural disorders of ear and mastoid process, not elsewhere classified  J300 Vasomotor rhinitis  J31 Chronic rhinitis, nasopharyngitis and pharyngitis  J32 Chronic sinusitis  J33 Nasal polyp  J341 Cyst and mucocele of nose and nasal sinus  J342 Deviated nasal septum  J343 Hypertrophy of nasal turbinates  J35 Chronic diseases of tonsils and adenoids  J37 Chronic laryngitis and laryngotracheitis  J380 Paralysis of vocal cords and larynx  J386 Stenosis of larynx  K051 Chronic gingivitis  K053 Chronic periodontitis  K07 Dentofacial anomalies [including malocclusion]  K110 Atrophy of salivary gland  K117 Disturbances of salivary secretion  Q30 Congenital malformations of nose  Q31 Congenital malformations of larynx  Q32 Congenital malformations of trachea and bronchus  Q35 Cleft palate  Q36 Cleft lip  Q37 Cleft palate with cleft lip  Q38 Other congenital malformations of tongue, mouth and pharynx |  |
| **Epilepsy** | G40 Epilepsy |  |
| **Esophagus, stomach and duodenum diseases** | I85 Oesophageal varices  I864 Gastric varices  I982 Oesophageal varices without bleeding in diseases classified elsewhere  I983 Oesophageal varices with bleeding in diseases classified elsewhere  K21 Gastro-oesophageal reflux disease  K220 Achalasia of cardia  K222 Oesophageal obstruction  K224 Dyskinesia of oesophagus  K225 Diverticulum of oesophagus, acquired  K227 Barrett oesophagus  K230 Tuberculous oesophagitis  K231 Megaoesophagus in Chagas disease  K254 Gastric ulcer: Chronic or unspecified with haemorrhage  K255 Gastric ulcer: Chronic or unspecified with perforation  K256 Gastric ulcer: Chronic or unspecified with both haemorrhage and perforation  K257 Gastric ulcer: Chronic without haemorrhage or perforation  K264 Duodenal ulcer: Chronic or unspecified with haemorrhage  K265 Duodenal ulcer: Chronic or unspecified with perforation  K266 Duodenal ulcer: Chronic or unspecified with both haemorrhage and perforation  K267 Duodenal ulcer: Chronic without haemorrhage or perforation  K274 Peptic ulcer, site unspecified: Chronic or unspecified with haemorrhage  K275 Peptic ulcer, site unspecified: Chronic or unspecified with perforation  K276 Peptic ulcer, site unspecified: Chronic or unspecified with both haemorrhage and perforation  K277 Peptic ulcer, site unspecified: Chronic without haemorrhage or perforation  K284 Gastrojejunal ulcer: Chronic or unspecified with haemorrhage  K285 Gastrojejunal ulcer: Chronic or unspecified with perforation  K286 Gastrojejunal ulcer: Chronic or unspecified with both haemorrhage and perforation  K287 Gastrojejunal ulcer: Chronic without haemorrhage or perforation  K293 Chronic superficial gastritis  K294 Chronic atrophic gastritis  K295 Chronic gastritis, unspecified  K296 Other gastritis  K297 Gastritis, unspecified  K298 Duodenitis  K299 Gastroduodenitis, unspecified  K311 Adult hypertrophic pyloric stenosis  K312 Hourglass stricture and stenosis of stomach  K313 Pylorospasm, not elsewhere classified  K314 Gastric diverticulum  K315 Obstruction of duodenum  Q39 Congenital malformations of oesophagus  Q40 Other congenital malformations of upper alimentary tract  Z903 Acquired absence of part of stomach | Use of other drugs for peptic ulcer and gastro-oesophageal reflux disease (A02BX) |
| **Glaucoma** | H401 Primary open-angle glaucoma  H402 Primary angle-closure glaucoma  H403 Glaucoma secondary to eye trauma  H404 Glaucoma secondary to eye inflammation  H405 Glaucoma secondary to other eye disorders  H406 Glaucoma secondary to drugs  H408 Other glaucoma  H409 Glaucoma, unspecified | Use of beta blocking agents (S01ED) |
| **Heart failure** | I110 Hypertensive heart disease with (congestive) heart failure  I130 Hypertensive heart and renal disease with (congestive) heart failure  I132 Hypertensive heart and renal disease with both (congestive) heart failure and renal failure  I27 Other pulmonary heart diseases  I280 Arteriovenous fistula of pulmonary vessels  I42 Cardiomyopathy  I43 Cardiomyopathy in diseases classified elsewhere  I50 Heart failure  I515 Myocardial degeneration  I517 Cardiomegaly  I528 Other heart disorders in other diseases classified elsewhere  Z941 Heart transplant status  Z943 Heart and lungs transplant status |  |
| **Hematological neoplasms** | C81 Hodgkin lymphoma  C82 Follicular lymphoma  C83 Non-follicular lymphoma  C84 Mature T/NK-cell lymphomas  C85 Other and unspecified types of non-Hodgkin lymphoma  C86 Other specified types of T/NK-cell lymphoma  C88 Malignant immunoproliferative diseases  C90 Multiple myeloma and malignant plasma cell neoplasms  C91 Lymphoid leukaemia  C92 Myeloid leukaemia  C93 Monocytic leukaemia  C94 Other leukaemias of specified cell type  C95 Leukaemia of unspecified cell type  C96 Other and unspecified malignant neoplasms of lymphoid, haematopoietic and related tissue |  |
| **Hypertension** | I10 Essential (primary) hypertension  I11 Hypertensive heart disease  I12 Hypertensive renal disease  I13 Hypertensive heart and renal disease  I15 Secondary hypertension | Blood pressure ≥140/90 mmHg^6^ |
| **Inflammatory arthropathies** | M023 Reiter disease  M05 Seropositive rheumatoid arthritis  M06 Other rheumatoid arthritis  M07 Psoriatic and enteropathic arthropathies  M08 Juvenile arthritis  M09 Juvenile arthritis in diseases classified elsewhere  M10 Gout  M11 Other crystal arthropathies  M12 Other specific arthropathies  M13 Other arthritis  M14 Arthropathies in other diseases classified elsewhere  M45 Ankylosing spondylitis  M460 Spinal enthesopathy  M461 Sacroiliitis, not elsewhere classified  M468 Other specified inflammatory spondylopathies  M469 Inflammatory spondylopathy, unspecified | Use of gold preparations (M01CB) |
| **Inflammatory bowel diseases** | K50 Crohn disease [regional enteritis]  K51 Ulcerative colitis | Use of intestinal antiinflammatory agents (A07E) |
| **Ischemic heart disease** | I20 Angina pectoris  I21 Acute myocardial infarction  I22 Subsequent myocardial infarction  I24 Other acute ischaemic heart diseases  I25 Chronic ischaemic heart disease  Z951 Presence of aortocoronary bypass graft  Z955 Presence of coronary angioplasty implant and graft | Use of organic nitrates (C01DA) or ranolazine (C01EB18) |
| **Migraine and facial pain syndromes** | G43 Migraine  G440 Cluster headache syndrome  G441 Vascular headache, not elsewhere classified  G442 Tension-type headache  G443 Chronic post-traumatic headache  G448 Other specified headache syndromes  G50 Disorders of trigeminal nerve | Use of antimigraine preparations (N02C) |
| **Multiple sclerosis** | G35 Multiple sclerosis |  |
| **Neurotic, stress-related and somatoform disorders** | F40 Phobic anxiety disorders  F41 Other anxiety disorders  F42 Obsessive-compulsive disorder  F43 Reaction to severe stress, and adjustment disorders  F44 Dissociative [conversion] disorders  F45 Somatoform disorders  F48 Other neurotic disorders |  |
| **Obesity** | E66 Obesity | Body Mass Index ≥30 kg/m^2^ |
| **Osteoarthrosis and other degenerative joint diseases** | M15 Polyarthrosis  M16 Coxarthrosis [arthrosis of hip]  M17 Gonarthrosis [arthrosis of knee]  M18 Arthrosis of first carpometacarpal joint  M19 Other arthrosis  M362 Haemophilic arthropathy  M363 Arthropathy in other blood disorders |  |
| **Osteoporosis** | M80 Osteoporosis with pathological fracture  M81 Osteoporosis without pathological fracture  M82 Osteoporosis in diseases classified elsewhere | Use of bisphosphonates (M05BA), bisphosphonate combinations (M05BB), strontium ranelate (M05BX03) or strontium ranelate and colecalciferol (M05BX53) |
| **Other cardiovascular diseases** | I09 Other rheumatic heart diseases  I281 Aneurysm of pulmonary artery  I310 Chronic adhesive pericarditis  I311 Chronic constrictive pericarditis  I456 Pre-excitation syndrome  I495 Sick sinus syndrome  I498 Other specified cardiac arrhythmias  I70 Atherosclerosis  I71 Aortic aneurysm and dissection  I72 Other aneurysm and dissection  I790 Aneurysm of aorta in diseases classified elsewhere  I791 Aortitis in diseases classified elsewhere  I950 Idiopathic hypotension  I951 Orthostatic hypotension  I958 Other hypotension  Q20 Congenital malformations of cardiac chambers and connections  Q21 Congenital malformations of cardiac septa  Q24 Other congenital malformations of heart  Q25 Congenital malformations of great arteries  Q26 Congenital malformations of great veins  Q27 Other congenital malformations of peripheral vascular system  Q28 Other congenital malformations of circulatory system  Z958 Presence of other cardiac and vascular implants and grafts  Z959 Presence of cardiac and vascular implant and graft, unspecified |  |
| **Other digestive disorders** | K660 Peritoneal adhesions  K900 Coeliac disease  K901 Tropical sprue  K902 Blind loop syndrome, not elsewhere classified  K911 Postgastric surgery syndromes  K93 Disorders of other digestive organs in diseases classified elsewhere  Q41 Congenital absence, atresia and stenosis of small intestine  Q42 Congenital absence, atresia and stenosis of large intestine  Q43 Other congenital malformations of intestine  R15 Faecal incontinence  Z904 Acquired absence of other parts of digestive tract  Z980 Intestinal bypass and anastomosis status |  |
| **Other eye diseases** | H022 Lagophthalmos  H023 Blepharochalasis  H024 Ptosis of eyelid  H025 Other disorders affecting eyelid function  H04 Disorders of lacrimal system  H05 Disorders of orbit  H104 Chronic conjunctivitis  H17 Corneal scars and opacities  H184 Corneal degeneration  H185 Hereditary corneal dystrophies  H186 Keratoconus  H187 Other corneal deformities  H188 Other specified disorders of cornea  H189 Disorder of cornea, unspecified  H193 Keratitis and keratoconjunctivitis in other diseases classified elsewhere  H198 Other disorders of sclera and cornea in diseases classified elsewhere  H201 Chronic iridocyclitis  H21 Other disorders of iris and ciliary body  H310 Chorioretinal scars  H311 Choroidal degeneration  H312 Hereditary choroidal dystrophy  H318 Other specified disorders of choroid  H319 Disorder of choroid, unspecified  H33 Retinal detachments and breaks  H352 Other proliferative retinopathy  H353 Degeneration of macula and posterior pole  H354 Peripheral retinal degeneration  H355 Hereditary retinal dystrophy  H357 Separation of retinal layers  H358 Other specified retinal disorders  H359 Retinal disorder, unspecified  H36 Retinal disorders in diseases classified elsewhere  H47 Other disorders of optic [2nd] nerve and visual pathways  H48 Disorders of optic [2nd] nerve and visual pathways in diseases classified elsewhere  H49 Paralytic strabismus  H51 Other disorders of binocular movement  Q10 Congenital malformations of eyelid, lacrimal apparatus and orbit  Q11 Anophthalmos, microphthalmos and macrophthalmos  Q13 Congenital malformations of anterior segment of eye  Q14 Congenital malformations of posterior segment of eye  Q15 Other congenital malformations of eye  Z947 Corneal transplant status |  |
| **Other genitourinary diseases** | B901 Sequelae of genitourinary tuberculosis  N200 Calculus of kidney  N202 Calculus of kidney with calculus of ureter  N209 Urinary calculus, unspecified  N210 Calculus in bladder  N218 Other lower urinary tract calculus  N219 Calculus of lower urinary tract, unspecified  N22 Calculus of urinary tract in diseases classified elsewhere  N301 Interstitial cystitis (chronic)  N302 Other chronic cystitis  N303 Trigonitis  N304 Irradiation cystitis  N31 Neuromuscular dysfunction of bladder, not elsewhere classified  N320 Bladder-neck obstruction  N323 Diverticulum of bladder  N328 Other specified disorders of bladder  N329 Bladder disorder, unspecified  N33 Bladder disorders in diseases classified elsewhere  N35 Urethral stricture  N393 Stress incontinence  N394 Other specified urinary incontinence  N480 Leukoplakia of penis  N484 Impotence of organic origin  N489 Disorder of penis, unspecified  N701 Chronic salpingitis and oophoritis  N711 Chronic inflammatory disease of uterus  N731 Chronic parametritis and pelvic cellulitis  N734 Female chronic pelvic peritonitis  N736 Female pelvic peritoneal adhesions  N761 Subacute and chronic vaginitis  N763 Subacute and chronic vulvitis  N81 Female genital prolapse  N88 Other noninflammatory disorders of cervix uteri  N895 Stricture and atresia of vagina  N905 Atrophy of vulva  N952 Postmenopausal atrophic vaginitis  Q54 Hypospadias  Q620 Congenital hydronephrosis  Q621 Atresia and stenosis of ureter  Q622 Congenital megaloureter  Q623 Other obstructive defects of renal pelvis and ureter  Q624 Agenesis of ureter  Q627 Congenital vesico-uretero-renal reflux  Q628 Other congenital malformations of ureter  Q638 Other specified congenital malformations of kidney  Q639 Congenital malformation of kidney, unspecified  Q640 Epispadias  Q641 Exstrophy of urinary bladder  Q643 Other atresia and stenosis of urethra and bladder neck  Q644 Malformation of urachus  Q645 Congenital absence of bladder and urethra  Q646 Congenital diverticulum of bladder  Q647 Other congenital malformations of bladder and urethra  Q648 Other specified congenital malformations of urinary system  Q649 Congenital malformation of urinary system, unspecified  Z906 Acquired absence of other organs of urinary tract  Z907 Acquired absence of genital organ(s)  Z960 Presence of urogenital implants |  |
| **Other metabolic diseases** | E20 Hypoparathyroidism  E21 Hyperparathyroidism and other disorders of parathyroid gland  E22 Hyperfunction of pituitary gland  E23 Hypofunction and other disorders of pituitary gland  E24 Cushing syndrome  E25 Adrenogenital disorders  E26 Hyperaldosteronism  E27 Other disorders of adrenal gland  E28 Ovarian dysfunction  E29 Testicular dysfunction  E31 Polyglandular dysfunction  E34 Other endocrine disorders  E35 Disorders of endocrine glands in diseases classified elsewhere  E40 Kwashiorkor  E41 Nutritional marasmus  E42 Marasmic kwashiorkor  E43 Unspecified severe protein-energy malnutrition  E44 Protein-energy malnutrition of moderate and mild degree  E45 Retarded development following protein-energy malnutrition  E46 Unspecified protein-energy malnutrition  E64 Sequelae of malnutrition and other nutritional deficiencies  E70 Disorders of aromatic amino-acid metabolism  E71 Disorders of branched-chain amino-acid metabolism and fatty-acid metabolism  E72 Other disorders of amino-acid metabolism  E74 Other disorders of carbohydrate metabolism  E75 Disorders of sphingolipid metabolism and other lipid storage disorders  E76 Disorders of glycosaminoglycan metabolism  E77 Disorders of glycoprotein metabolism  E79 Disorders of purine and pyrimidine metabolism  E80 Disorders of porphyrin and bilirubin metabolism  E83 Disorders of mineral metabolism  E84 Cystic fibrosis  E85 Amyloidosis  E88 Other metabolic disorders  E89 Postprocedural endocrine and metabolic disorders, not elsewhere classified  K903 Pancreatic steatorrhoea  K904 Malabsorption due to intolerance, not elsewhere classified  K908 Other intestinal malabsorption  K909 Intestinal malabsorption, unspecified  K912 Postsurgical malabsorption, not elsewhere classified  M83 Adult osteomalacia  M88 Paget disease of bone [osteitis deformans]  N25 Disorders resulting from impaired renal tubular function |  |
| **Other musculoskeletal and joint diseases** | B902 Sequelae of tuberculosis of bones and joints  M212 Flexion deformity  M213 Wrist or foot drop (acquired)  M214 Flat foot [pes planus] (acquired)  M215 Acquired clawhand, clubhand, clawfoot and clubfoot  M216 Other acquired deformities of ankle and foot  M217 Unequal limb length (acquired)  M218 Other specified acquired deformities of limbs  M219 Acquired deformity of limb, unspecified  M22 Disorders of patella  M23 Internal derangement of knee  M24 Other specific joint derangements  M252 Flail joint  M253 Other instability of joint  M357 Hypermobility syndrome  M61 Calcification and ossification of muscle  M652 Calcific tendinitis  M653 Trigger finger  M654 Radial styloid tenosynovitis [de Quervain]  M700 Chronic crepitant synovitis of hand and wrist  M720 Palmar fascial fibromatosis [Dupuytren]  M722 Plantar fascial fibromatosis  M724 Pseudosarcomatous fibromatosis  M750 Adhesive capsulitis of shoulder  M751 Rotator cuff syndrome  M753 Calcific tendinitis of shoulder  M754 Impingement syndrome of shoulder  M797 Fibromyalgia  M841 Nonunion of fracture [pseudarthrosis]  M89 Other disorders of bone  M91 Juvenile osteochondrosis of hip and pelvis  M93 Other osteochondropathies  M94 Other disorders of cartilage  M96 Postprocedural musculoskeletal disorders, not elsewhere classified  M99 Biomechanical lesions, not elsewhere classified  Q65 Congenital deformities of hip  Q66 Congenital deformities of feet  Q68 Other congenital musculoskeletal deformities  Q71 Reduction defects of upper limb  Q72 Reduction defects of lower limb  Q73 Reduction defects of unspecified limb  Q74 Other congenital malformations of limb(s)  Q77 Osteochondrodysplasia with defects of growth of tubular bones and spine  Q78 Other osteochondrodysplasias  Q796 Ehlers-Danlos syndrome  Q798 Other congenital malformations of musculoskeletal system  Q87 Other specified congenital malformation syndromes affecting multiple systems  S382 Traumatic amputation of external genital organs  S48 Traumatic amputation of shoulder and upper arm  S58 Traumatic amputation of forearm  S68 Traumatic amputation of wrist and hand  S78 Traumatic amputation of hip and thigh  S88 Traumatic amputation of lower leg  S98 Traumatic amputation of ankle and foot  T05 Traumatic amputations involving multiple body regions  T096 Traumatic amputation of trunk, level unspecified  T116 Traumatic amputation of upper limb, level unspecified  T136 Traumatic amputation of lower limb, level unspecified  T147 Crushing injury and traumatic amputation of unspecified body region  T90 Sequelae of injuries of head  T91 Sequelae of injuries of neck and trunk  T92 Sequelae of injuries of upper limb  T93 Sequelae of injuries of lower limb  T94 Sequelae of injuries involving multiple and unspecified body regions  T95 Sequelae of burns, corrosions and frostbite  T96 Sequelae of poisoning by drugs, medicaments and biological substances  T97 Sequelae of toxic effects of substances chiefly nonmedicinal as to source  T98 Sequelae of other and unspecified effects of external causes  Z440 Fitting and adjustment of artificial arm (complete)(partial)  Z441 Fitting and adjustment of artificial leg (complete)(partial)  Z891 Acquired absence of hand and wrist  Z892 Acquired absence of upper limb above wrist  Z893 Acquired absence of both upper limbs [any level]  Z894 Acquired absence of foot and ankle  Z895 Acquired absence of leg at or below knee  Z896 Acquired absence of leg above knee  Z897 Acquired absence of both lower limbs [any level, except toes alone]  Z898 Acquired absence of upper and lower limbs [any level]  Z899 Acquired absence of limb, unspecified  Z946 Bone transplant status  Z966 Presence of orthopaedic joint implants  Z971 Presence of artificial limb (complete)(partial) |  |
| **Other neurological diseases** | B900 Sequelae of central nervous system tuberculosis  D482 Neoplasm of uncertain or unknown behaviour: Peripheral nerves and autonomic nervous system  G041 Tropical spastic paraplegia  G09 Sequelae of inflammatory diseases of central nervous system  G10 Huntington disease  G11 Hereditary ataxia  G12 Spinal muscular atrophy and related syndromes  G13 Systemic atrophies primarily affecting central nervous system in diseases classified elsewhere  G24 Dystonia  G25 Other extrapyramidal and movement disorders  G26 Extrapyramidal and movement disorders in diseases classified elsewhere  G32 Other degenerative disorders of nervous system in diseases classified elsewhere  G37 Other demyelinating diseases of central nervous system  G51 Facial nerve disorders  G52 Disorders of other cranial nerves  G53 Cranial nerve disorders in diseases classified elsewhere  G70 Myasthenia gravis and other myoneural disorders  G71 Primary disorders of muscles  G723 Periodic paralysis  G724 Inflammatory myopathy, not elsewhere classified  G728 Other specified myopathies  G729 Myopathy, unspecified  G73 Disorders of myoneural junction and muscle in diseases classified elsewhere  G80 Cerebral palsy  G81 Hemiplegia  G82 Paraplegia and tetraplegia  G83 Other paralytic syndromes  G90 Disorders of autonomic nervous system  G91 Hydrocephalus  G938 Other specified disorders of brain  G939 Disorder of brain, unspecified  G95 Other diseases of spinal cord  G99 Other disorders of nervous system in diseases classified elsewhere  M471 Other spondylosis with myelopathy  Q00 Anencephaly and similar malformations  Q01 Encephalocele  Q02 Microcephaly  Q03 Congenital hydrocephalus  Q04 Other congenital malformations of brain  Q05 Spina bifida  Q06 Other congenital malformations of spinal cord  Q07 Other congenital malformations of nervous system  Q760 Spina bifida occulta |  |
| **Other psychiatric and behavioral diseases** | F04 Organic amnesic syndrome, not induced by alcohol and other psychoactive substances  F06 Other mental disorders due to brain damage and dysfunction and to physical disease  F07 Personality and behavioural disorders due to brain disease, damage and dysfunction  F09 Unspecified organic or symptomatic mental disorder  F102 Mental and behavioural disorders due to use of alcohol: Dependence syndrome  F106 Mental and behavioural disorders due to use of alcohol: Amnesic syndrome  F107 Mental and behavioural disorders due to use of alcohol: Residual and late-onset psychotic disorder  F112 Mental and behavioural disorders due to use of opioids: Dependence syndrome  F116 Mental and behavioural disorders due to use of opioids: Amnesic syndrome  F117 Mental and behavioural disorders due to use of opioids: Residual and late-onset psychotic disorder  F122 Mental and behavioural disorders due to use of cannabinoids: Dependence syndrome  F126 Mental and behavioural disorders due to use of cannabinoids: Amnesic syndrome  F127 Mental and behavioural disorders due to use of cannabinoids: Residual and late-onset psychotic disorder  F132 Mental and behavioural disorders due to use of sedatives or hypnotics: Dependence syndrome  F136 Mental and behavioural disorders due to use of sedatives or hypnotics: Amnesic syndrome  F137 Mental and behavioural disorders due to use of sedatives or hypnotics: Residual and late-onset psychotic disorder  F142 Mental and behavioural disorders due to use of cocaine: Dependence syndrome  F146 Mental and behavioural disorders due to use of cocaine: Amnesic syndrome  F147 Mental and behavioural disorders due to use of cocaine: Residual and late-onset psychotic disorder  F152 Mental and behavioural disorders due to use of other stimulants, including caffeine: Dependence syndrome  F156 Mental and behavioural disorders due to use of other stimulants, including caffeine: Amnesic syndrome  F157 Mental and behavioural disorders due to use of other stimulants, including caffeine: Residual and late-onset psychotic disorder  F162 Mental and behavioural disorders due to use of hallucinogens: Dependence syndrome  F166 Mental and behavioural disorders due to use of hallucinogens: Amnesic syndrome  F167 Mental and behavioural disorders due to use of hallucinogens: Residual and late-onset psychotic disorder  F172 Mental and behavioural disorders due to use of tobacco: Dependence syndrome  F176 Mental and behavioural disorders due to use of tobacco: Amnesic syndrome  F177 Mental and behavioural disorders due to use of tobacco: Residual and late-onset psychotic disorder  F182 Mental and behavioural disorders due to use of volatile solvents: Dependence syndrome  F186 Mental and behavioural disorders due to use of volatile solvents: Amnesic syndrome  F187 Mental and behavioural disorders due to use of volatile solvents: Residual and late-onset psychotic disorder  F192 Mental and behavioural disorders due to multiple drug use and use of other psychoactive substances: Dependence syndrome  F196 Mental and behavioural disorders due to multiple drug use and use of other psychoactive substances: Amnesic syndrome  F197 Mental and behavioural disorders due to multiple drug use and use of other psychoactive substances: Residual and late-onset psychotic disorder  F50 Eating disorders  F52 Sexual dysfunction, not caused by organic disorder or disease  F60 Specific personality disorders  F61 Mixed and other personality disorders  F62 Enduring personality changes, not attributable to brain damage and disease  F63 Habit and impulse disorders  F68 Other disorders of adult personality and behaviour  F70 Mild mental retardation  F71 Moderate mental retardation  F72 Severe mental retardation  F73 Profound mental retardation  F78 Other mental retardation  F79 Unspecified mental retardation  F80 Specific developmental disorders of speech and language  F81 Specific developmental disorders of scholastic skills  F82 Specific developmental disorder of motor function  F83 Mixed specific developmental disorders  F84 Pervasive developmental disorders  F88 Other disorders of psychological development  F89 Unspecified disorder of psychological development  F95 Tic disorders  F99 Mental disorder, not otherwise specified | Use of drugs for alcohol dependence (N07BB) |
| **Other respiratory diseases** | B909 Sequelae of respiratory and unspecified tuberculosis  E662 Extreme obesity with alveolar hypoventilation  J60 Coalworker pneumoconiosis  J61 Pneumoconiosis due to asbestos and other mineral fibres  J62 Pneumoconiosis due to dust containing silica  J63 Pneumoconiosis due to other inorganic dusts  J64 Unspecified pneumoconiosis  J65 Pneumoconiosis associated with tuberculosis  J66 Airway disease due to specific organic dust  J67 Hypersensitivity pneumonitis due to organic dust  J684 Chronic respiratory conditions due to chemicals, gases, fumes and vapours  J701 Chronic and other pulmonary manifestations due to radiation  J703 Chronic drug-induced interstitial lung disorders  J704 Drug-induced interstitial lung disorders, unspecified  J84 Other interstitial pulmonary diseases  J92 Pleural plaque  J941 Fibrothorax  J953 Chronic pulmonary insufficiency following surgery  J955 Postprocedural subglottic stenosis  J961 Chronic respiratory failure  J98 Other respiratory disorders  Q33 Congenital malformations of lung  Q34 Other congenital malformations of respiratory system  Z902 Acquired absence of lung [part of]  Z942 Lung transplant status  Z943 Heart and lungs transplant status  Z963 Presence of artificial larynx |  |
| **Other skin diseases** | L13 Other bullous disorders  L28 Lichen simplex chronicus and prurigo  L301 Dyshidrosis [pompholyx]  L43 Lichen planus  L508 Other urticaria  L581 Chronic radiodermatitis  L85 Other epidermal thickening  Q80 Congenital ichthyosis  Q81 Epidermolysis bullosa  Q821 Xeroderma pigmentosum  Q822 Mastocytosis  Q829 Congenital malformation of skin, unspecified |  |
| **Parkinson and parkinsonism** | G20 Parkinson disease  G21 Secondary parkinsonism  G22 Parkinsonism in diseases classified elsewhere  G23 Other degenerative diseases of basal ganglia | Use of dopa and dopa derivatives (N04BA), dopamine agonists (N04BC), or other dopaminergic agents (N04BX) |
| **Peripheral neuropathy** | B91 Sequelae of poliomyelitis  G14 Postpolio syndrome  G54 Nerve root and plexus disorders  G55 Nerve root and plexus compressions in diseases classified elsewhere  G56 Mononeuropathies of upper limb  G57 Mononeuropathies of lower limb  G58 Other mononeuropathies  G59 Mononeuropathy in diseases classified elsewhere  G60 Hereditary and idiopathic neuropathy  G628 Other specified polyneuropathies  G629 Polyneuropathy, unspecified  G63 Polyneuropathy in diseases classified elsewhere  M472 Other spondylosis with radiculopathy  M531 Cervicobrachial syndrome  M541 Radiculopathy |  |
| **Peripheral vascular disease** | I702 Atherosclerosis of arteries of extremities  I73 Other peripheral vascular diseases  I792 Peripheral angiopathy in diseases classified elsewhere  I798 Other disorders of arteries, arterioles and capillaries in diseases classified elsewhere | Use of cilostazol (B01AC23) |
| **Prostate diseases** | N40 Hyperplasia of prostate  N411 Chronic prostatitis  N418 Other inflammatory diseases of prostate | Use of drugs for benign prostatic hypertrophy excluding testosterone-5-alpha reductase inhibitors (G04C excl. G04CB) |
| **Schizophrenia and delusional diseases** | F20 Schizophrenia  F22 Persistent delusional disorders  F24 Induced delusional disorder  F25 Schizoaffective disorders  F28 Other nonorganic psychotic disorders |  |
| **Sleep disorders** | F510 Nonorganic insomnia  F511 Nonorganic hypersomnia  F512 Nonorganic disorder of the sleep-wake schedule  F513 Sleepwalking [somnambulism]  G47 Sleep disorders |  |
| **Solid neoplasms** | C Malignant neoplasms  D00 Carcinoma in situ of oral cavity, oesophagus and stomach  D01 Carcinoma in situ of other and unspecified digestive organs  D02 Carcinoma in situ of middle ear and respiratory system  D03 Melanoma in situ  D04 Carcinoma in situ of skin  D05 Carcinoma in situ of breast  D06 Carcinoma in situ of cervix uteri  D07 Carcinoma in situ of other and unspecified genital organs  D09 Carcinoma in situ of other and unspecified sites  D320 Benign neoplasm: Cerebral meninges  D321 Benign neoplasm: Spinal meninges  D329 Benign neoplasm: Meninges, unspecified  D330 Benign neoplasm: Brain, supratentorial  D331 Benign neoplasm: Brain, infratentorial  D332 Benign neoplasm: Brain, unspecified  D333 Benign neoplasm: Cranial nerves  D334 Benign neoplasm: Spinal cord  Q85 Phakomatoses, not elsewhere classified |  |
| **Thyroid diseases** | E00 Congenital iodine-deficiency syndrome  E01 Iodine-deficiency-related thyroid disorders and allied conditions  E02 Subclinical iodine-deficiency hypothyroidism  E03 Other hypothyroidism  E05 Thyrotoxicosis [hyperthyroidism]  E062 Chronic thyroiditis with transient thyrotoxicosis  E063 Autoimmune thyroiditis  E065 Other chronic thyroiditis  E07 Other disorders of thyroid  E350 Disorders of thyroid gland in diseases classified elsewhere  E890 Postprocedural hypothyroidism | Use of thyroid hormones (H03AA) or antithyroid preparations (H03B) |
| **Venous and lymphatic diseases** | I780 Hereditary haemorrhagic telangiectasia  I83 Varicose veins of lower extremities  I87 Other disorders of veins  I89 Other noninfective disorders of lymphatic vessels and lymph nodes  I972 Postmastectomy lymphoedema syndrome  Q820 Hereditary lymphoedema |  |

^*^The ATC codes corresponding to each drug are shown in brackets. Only drugs that can be unequivocally linked to chronic conditions were considered. Drugs with more than one indication were excluded from the list. The selection of ATC codes was based on a literature review and the clinical judgement of physicians. The criteria presented in this table were used in addition to the diagnoses assigned in SNAC-K.

# **Appendix 4****. Association between seasonality and multimorbidity patterns in LRTI-associated hospitalizations and all-cause 30-day readmissions**


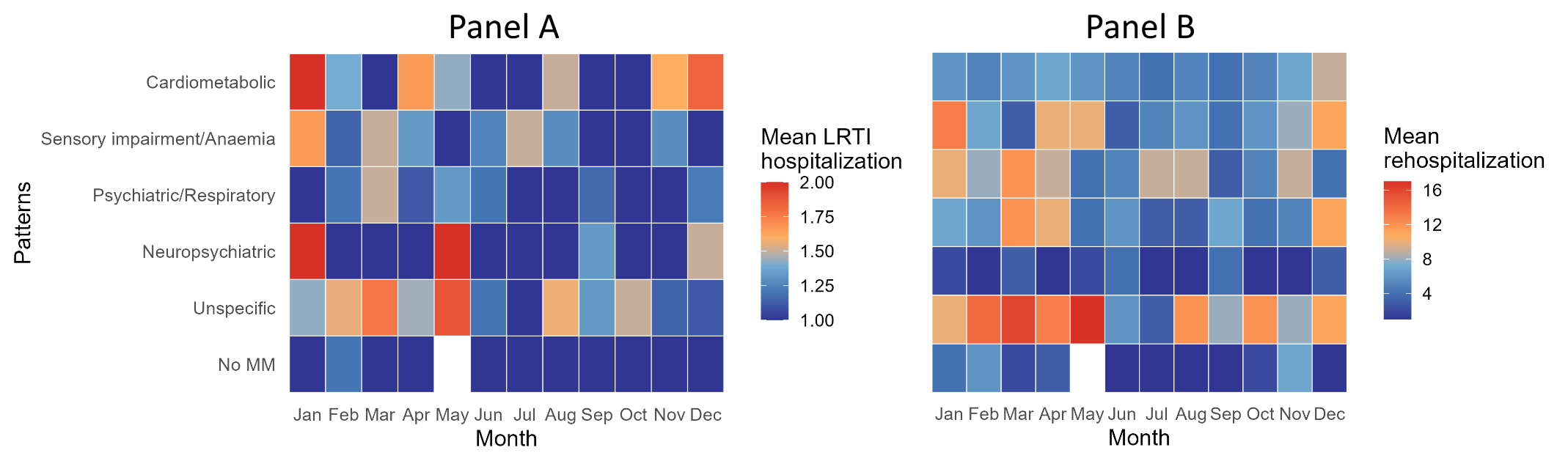


Heatmaps display monthly trends (January–December) across multimorbidity patterns. In **Panel A**, LRTI-associated hospitalization by month, while in **Panel B,** mean all-cause 30-day readmissions. Overall, the figure illustrates heterogeneity in both hospitalization and rehospitalization across multimorbidity patterns and calendar months, with evidence of seasonal fluctuation and consistently lower burden among individuals without multimorbidity.

# **Appendix 5****. Hazard of hospital readmission within 120 days following first LRTI across multimorbidity patterns**


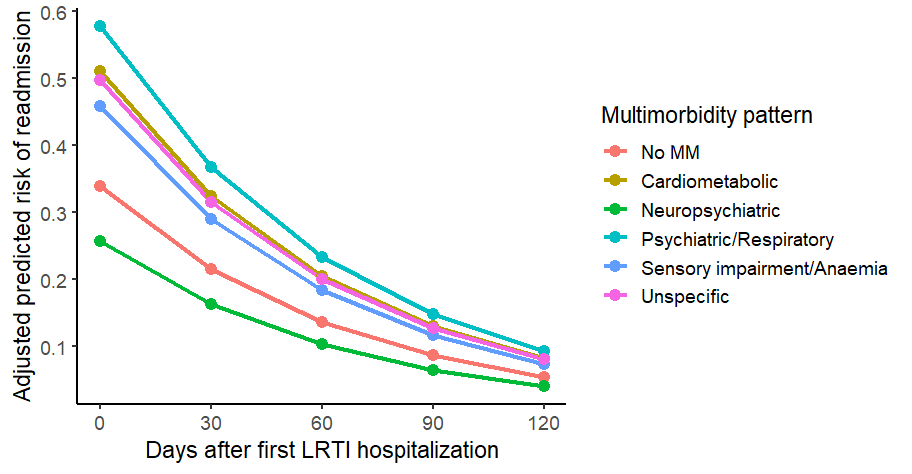


Adjusted predicted cumulative hazard of hospital readmission, after a first LRTI-associated hospitalization, at 30, 60, 90, and 120 days, stratified by multimorbidity pattern. Estimates were derived from a Poisson regression model with log link using generalized estimating equations (GEE) and an exchangeable correlation structure to account for repeated measurements within individuals. Models were adjusted for age, sex, length of stay, educational level, civil status, smoking status, walking speed, and disability.

# **Appendix 6****. Sensitivity analysis per age, sex, walking speed, disability, institutionalization and LRTI-associated 30-day rehospitalization**

|  | ***Multimorbidity patterns*** | | | | | |
| --- | --- | --- | --- | --- | --- | --- |
|  | **No Multimorbidity** | **Unspeficic** | **Sensory impairment/Anaemia** | **Psychiatric/Respiratory** | **Cardiometabolic** | **Neuropsychiatric** |
|  | HR (95% CI) | HR (95% CI) | HR (95% CI) | HR (95% CI) | HR (95% CI) | HR (95% CI) |
| *Age* |  |  |  |  |  |  |
| Under 78 | REF | 1.15  (0.70;1.90) | **2.54**  **(1.31; 4.95)** | **2.23**  **(1.28; 3.89)** | **4.44**  **(2.17; 9.11)** | **12.61**  **(3.60; 44.21)** |
| Over 78 | REF | 0.79  (0.38;1.64) | 0.84  (0.40; 1.76) | 1.10  (0.51; 2.37) | 1.99  (0.95; 4.20) | 1.08  (0.44; 2.65) |
| *Sex* |  |  |  |  |  |  |
| Female | REF | 0.88  (0.50;1.57) | 1.01  (0.54; 1.89) | 1.48  (0.82 ;2.68) | **2.52**  **(1.31; 4.82)** | 1.06  (0.46; 2.45) |
| Male | REF | 1.27  (0.70;2.28) | 1.53  (0.77; 3.04) | **2.21**  **(1.11; 4.39)** | **3.25**  **(1.64; 6.43)** | **4.38**  **(1.50; 12.77)** |
| *Walking speed* |  |  |  |  |  |  |
| >0.8m/s | REF | 1.09  (0.70;1.71) | 1.18  (0.69; 2.01) | **1.89**  **(1.16; 3.10)** | **2.78**  **(1.57; 4.92)** | 1.31  (0.30; 5.84) |
| ≤0.8m/s | REF | 0.51  (0.17;1.48) | 0.68  (0.24; 1.92) | 0.84  (0.29; 2.43) | 1.48  (0.53; 4.15) | 0.88  (0.28; 2.70) |
| *Disability* |  |  |  |  |  |  |
| No impairment | REF | 1.07  (0.71;1.62) | 1.20  (0.75; 1.92) | **1.82**  **(1.16; 2.83)** | **2.92**  **(1.82; 4.68)** | 1.89  (0.92; 3.88) |
| 1+ impairment | REF | 1.10  (0.73;1.65) | 1.24  (0.78; 1.97) | **1.89**  **(1.21; 2.94)** | **3.22**  **(2.03; 5.13)** | **2.06**  **(1.01; 4.21)** |
| *Institutionalization* |  |  |  |  |  |  |
| Yes | REF | NA | 1.46  (0.04; 49.61) | 9.40  (0.19; 455.71) | 1.35  (0.04; 49.97) | 0.82  (0.03; 21.45) |
| No | REF | 1.08  (0.71;1.62) | 1.23  (0.78; 1.96) | **1.81**  **(1.16; 2.82)** | **2.90**  **(1.82; 4.64)** | 1.76  (0.90; 3.45) |
|  | ***Multimorbidity patterns*** | | | | | |
|  | **No Multimorbidity** | **Unspeficic** | **Sensory impairment/Anaemia** | **Psychiatric/Respiratory** | **Cardiometabolic** | **Neuropsychiatric** |
|  | HR (95% CI) | HR (95% CI) | HR (95% CI) | HR (95% CI) | HR (95% CI) | HR (95% CI) |
| *Age* |  |  |  |  |  |  |
| Under 78 | REF | 1.92  (0.57; 6.39) | **6.16**  **(1.13; 33.64)** | **4.75**  **(1.16; 19.39)** | 3.29  (0.79; 13.76) | 4.46  (0.08; 237.82) |
| Over 78 | REF | NA | 1.25  (0.66; 2.38) | 1.14  (0.53; 2.45) | 2.20  (0.99; 4.89) | 2.51  (0.73; 8.68) |
| *Sex* |  |  |  |  |  |  |
| Female | REF | 1.63  (0.35; 7.68) | 0.98  (0.19; 5.03) | 2.06  (0.42; 10.10) | 2.09  (0.38; 11.32) | 1.46  (0.18; 11.74) |
| Male | REF | 1.50  (0.37; 6.04) | 5.27  (0.99; 27.91) | 3.49  (0.68; 18.02) | **6.39**  **(1.20; 33.93)** | 7.02  (0.87; 56.37) |
| *Walking speed* |  |  |  |  |  |  |
| >0.8m/s | REF | 2.51  (0.80; 7.92) | 2.01  (0.55; 7.38) | **4.09**  **(1.24; 13.50)** | **5.70**  **(1.59; 20.39)** | NA |
| ≤0.8m/s | REF | 0.03  (0.00; 0.65) | 0.07  (0.00; 1.06) | 0.05  (0.00; 0.99) | 0.09  (0.01; 1.25) | 0.13  (0.01; 2.14) |
| *Disability* |  |  |  |  |  |  |
| No impairment | REF | 1.38  (0.51; 3.74) | 1.42  (0.48; 4.21) | 2.29  (0.82; 6.43) | **3.35**  **(1.14; 9.89)** | **4.77**  **(1.10; 20.75)** |
| *Institutionalization* |  |  |  |  |  |  |
| No | REF | 1.32  (0.49; 3.55) | 1.36  (0.47; 3.93) | 1.89  (0.68; 5.24) | **3.14**  **(1.08; 9.13)** | **4.09**  **(1.01; 16.63)** |

CI: confidence interval; HR: hazard ratio; NA: not evaluable

Models were adjusted for sex, age, education status, civil status, smoking status, walking speed and disability and LoS. The model was rank deficient due to low numerosity; thus, it was not possible to perform the sensitivity analysis for all-cause 30-day readmissions in institutionalized participants and participants with impairment.

# **Appendix 7****. Analysis on LRTI-associated 30-day readmissions**

| **LRTI-associated 30-day readmissions** | | | |
| --- | --- | --- | --- |
|  | **Hospitalized/**  **Total** | **Incidence rate** | **Cox model** |
|  |  | Per 100 person-years | HR (95% CI) |
| *Multimorbidity patterns* |  |  |  |
| No Multimorbidity | 22/412 | 0.32 | REF |
| Unspecific | 101/1259 | 0.21 | 1.45  (0.48; 4.36) |
| Sensory impairment/ Anaemia | 79/567 | 0.33 | 1.60  (0.50; 5.15) |
| Psychiatric/ Respiratory | 68/468 | 0.31 | 1.89  (0.61; 5.85) |
| Cardiometabolic | 77/330 | 0.45 | **3.36**  **(1.04; 10.84)** |
| Neuropsychiatric | 18/265 | 1.00 | 2.73  (0.52; 14.24) |

CI: confidence interval; HR: hazard ratio.

Models were adjusted for sex, age, education status, civil status, smoking status, walking speed and disability and LoS, for all-cause 30-day readmissions.

#

#

# 
